# Ultra-Sensitive Flexible Tactile Sensor Based on Graphene Film

**DOI:** 10.3390/mi10110730

**Published:** 2019-10-28

**Authors:** Xiaozhou Lü, Liang Qi, Hanlun Hu, Xiaoping Li, Guanghui Bai, Jun Chen, Weimin Bao

**Affiliations:** 1 School of Aerospace Science and Technology, Xidian University, Xi’an 710071, China; lqi_1@stu.xidian.edu.cn (L.Q.); huhanluna@163.com (H.H.); xpli@xidian.edu.cn (X.L.); baoweimin@cashq.ac.cn (W.B.); 2 Science and Technology on Space Physics Laboratory, Beijing 100076, China; 3 China Academy of Launch Vehicle Technology, Beijing 100076, China; bghbuaa@aliyun.com

**Keywords:** flexible tactile sensors, ultra-sensitive, graphene film

## Abstract

Flexible tactile sensor can be integrated into artificial skin and applied in industrial robot and biomedical engineering. However, the presented tactile sensors still have challenge in increasing sensitivity to expand the sensor’s application. Aiming at this problem, this paper presents an ultra-sensitive flexible tactile sensor. The sensor is based on piezoresistive effect of graphene film and is composed of upper substrate (PDMS bump with a size of 5 mm × 7 mm and a thickness of 1 mm), medial Graphene/PET film (Graphene/PET film with a size of 5 mm × 7 mm, PET with a hardness of 2H) and lower substrate (PI with fabricated electrodes). We presented the structure and reduced the principle of the sensor. We also fabricated several sample devices of the sensor and carried out experiment to test the performance. The results show that the sensor performed an ultra high sensitivity of 10.80/kPa at the range of 0–4 kPa and have a large measurement range up to 600 kPa. The sensor has 4 orders of magnitude between minimum resolution and maximum measurement range which have great advantage compared with state of the art. The sensor is expected to have great application prospect in robot and biomedical.

## 1. Introduction

Flexible tactile sensor is an electronic device which is able to mimics human skin tactile receptors. It can be integrated into artificial skin to get pressure information from external stimulation. With the development of artificial skin, flexible tactile sensor has been widely applied to industrial robot and biomedical engineering [1] which requires the flexible tactile sensor have ultra high sensitivity to sense micro-pressure.

Presented flexible tactile sensors can be classified into three types according to the working principle which are capacitive [2,3,4], piezoelectric [5,6,7] and piezoresistive [8,9,10,11,12,13].

Capacitive flexible tactile sensors are using to measure micro-pressure according to the capacitance varied with the compressed elastic material between upper and lower electrodes [2,3,4]. It has the advantages of high sensitivity, quick response, temperature insensitive and convenience of flexible design [4]. However, the sensitivity of capacitive tactile sensor is fairly ordinary (typical 0.004 kPa${}^{-1}$) [14] and the parasitic capacitances cased by measuring object is difficult to avoid and eliminate.

Piezoelectric tactile sensors are using to measure micro-pressure according to the piezoelectric effect of flexible piezoelectric materials such as PVDF [5], PZT [6] and ZnO piezoelectric nanowires [7]. Piezoelectric tactile sensor has a excellent dynamic performance but ordinary static linearity performance. For example, Witing Liu et al. [5] presented a fingertip piezoelectric tactile sensor array which is working in high frequency (400 Hz) to measure the surface texture of objects.

Piezoresistive tactile sensors are using to measure the micro-pressure according to piezoresistive effect of flexible resistive materials. Andrea Rinaldi et al. [15] presented a pressure sensor based on Graphene Nanoplatelets. The sensor have a sensitivity of 0.23 kPa${}^{-1}$ and a measurement resolution of 1 Pa. However, the sensor have a thickness of 4 mm which great restrict the application on tactile sensor. Youngdo Jung et al. [16] presented a tactile sensor based on composite material of CNTs/PDMS. The sensor have sensitivity of 0.10173 kPa${}^{-1}$ at a range of 0.128–44 kPa. However, this sensor have a size of 15 mm × 15 mm × 5 mm which still have challenge in miniaturization to apply on robot hand.

Therefore, the presented tactile sensors have a high sensitivity but small measurement range or have a large measurement range but low sensitivity. The measurement range and the sensitivity are a pair of contradiction. For example, the presented sensors always have 2–3 orders of magnitude between minimum resolution and maximum measurement range which greatly limited the application in robot hands.

To aiming at the above problem, this paper presented an ultra-sensitivity flexible tactile sensor based on piezoresistive effect of graphene film. The sensor is composed of upper substrate (PDMS), medial film (Graphene/PET) and lower substrate (PI) and is expected to have an ultra high sensitivity and have an excellent characteristic of miniaturization and flexibility. The sensor also performs excellently in sensitivity and measurement range which has 4 orders of magnitude between minimum resolution and maximum measurement range. The sensor may greatly expand the application of flexible tactile sensor in industrial robot and biomedical engineering.

## 2. Method

**Figure 1:**
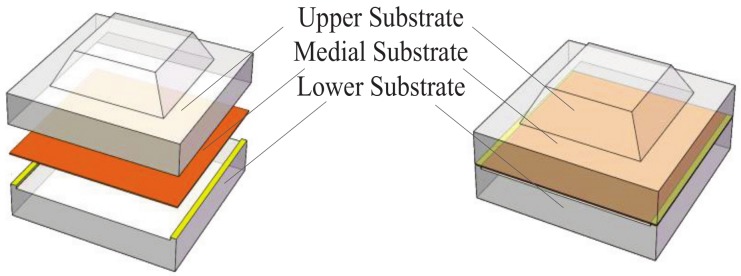
The structure of the flexible tactile sensor.

The structure of the flexible tactile sensor presented in this work is shown as in Figure 1. The sensor was composed of three layers which are upper substrate, medial graphene/PET film and lower substrate. The prepared graphene film was transferred to the PET substrate. A bump was fabricated on the upper substrate to collect and focus stress to the graphene film.

**Figure 2:**
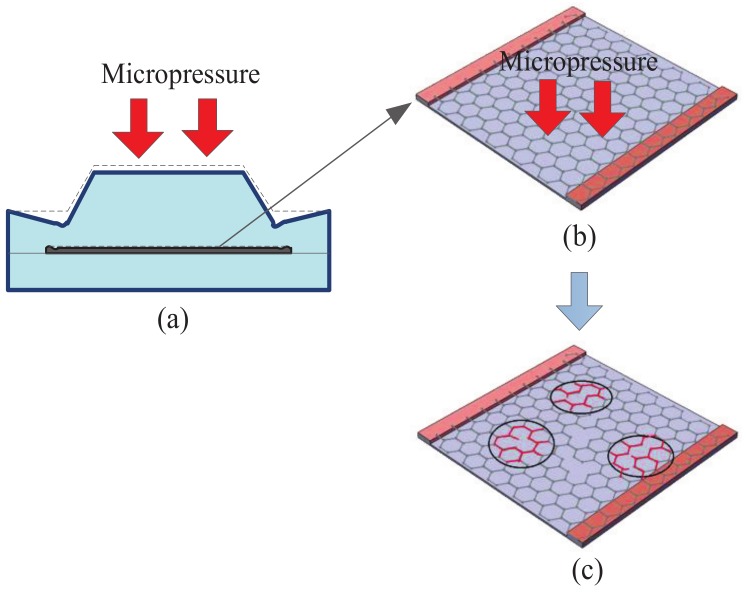
Schematic diagram of working principle of tactile sensor; (**a**) Schematic diagram of tactile sensor subjected to micro-pressure; (**b**) Morphology of graphene thin films before being subjected to micro-pressure; (**c**) Microstructure of graphene film under compression.

The principle of the flexible tactile sensor is piezoresistive effect of the graphene/PET film. When a micro-pressure is applied on the surface of the sensor, the upper bump will collect the stress evenly to the graphene film (see Figure 2a,b). These stress will make the C-C bond of the graphene film fractured or cracked (see Figure 2c) and the resistivity of the graphene film changed. Therefore, we can measure the applied micro-pressure according to the varied resistance of the graphene film. Benefiting of the excellent sensitivity and flexibility of graphene film, the sensor will have an ultra-high sensitivity.

## 3. Material

The flexible tactile sensor presented in this work was composed of three layers which is upper substrate, medial graphene film and lower substrate. The upper substrate was made of polydimethylsiloxane (PDMS) which have excellent features of super-elastic, easy processing and chemical stability. The medial sensing film was made of single-layer graphene transferred on Polyethylene terephthalate (PET) film which have features of excellent sensitivity and flexibility. The lower substrate was made of Polyimide (PI) which is flexible and easy to fabricate electrodes.

**Figure 3:**
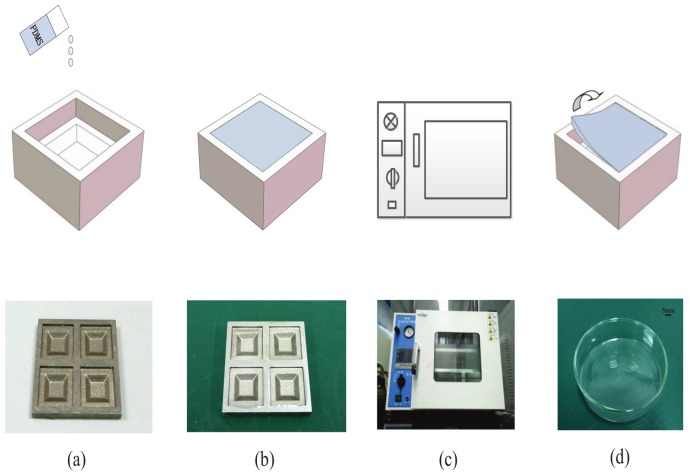
The fabrication process of upper substrate; (**a**) A stainless steel negative mold was fabricated; (**b**) The silicone elastomer base and elastomer curing of PDMS with a rate of 10:1 was fully stirred and then poured into the stainless steel negative mold; (**c**) Dry the PDMS for 4 h in a a vacuum dryer ar the temperature to 80 ${}^{\circ }$ C; (**d**) Remove the upper substrate from the mold.

The upper PDMS substrate was fabricated by a casting method. The specific fabricating process was shown as in Figure 3. Firstly, we fabricated a stainless steel negative mold which is best fitting the size of the upper substrate (see Figure 3a). Secondly, we mixed the silicone elastomer base and elastomer curing of PDMS (SYLGARD 184, Dow Corning Co., Midland, MI, USA) with a rate of 10:1, and put the mixture PDMS into the stainless steel mold (see Figure 3b). Thirdly, we put the stainless steel mold with mixture PDMS into a vacuum drying oven (LiChen, DZF) for 4 h at a temperature of 80 (see Figure 3c). At last, we peeled off the upper PDMS substrate from the stainless steel mold (see Figure 3d). If the different shape and size of stainless steel mold was prepared, the different shape and size upper PDMS substrate would be fabricated.

**Figure 4:**
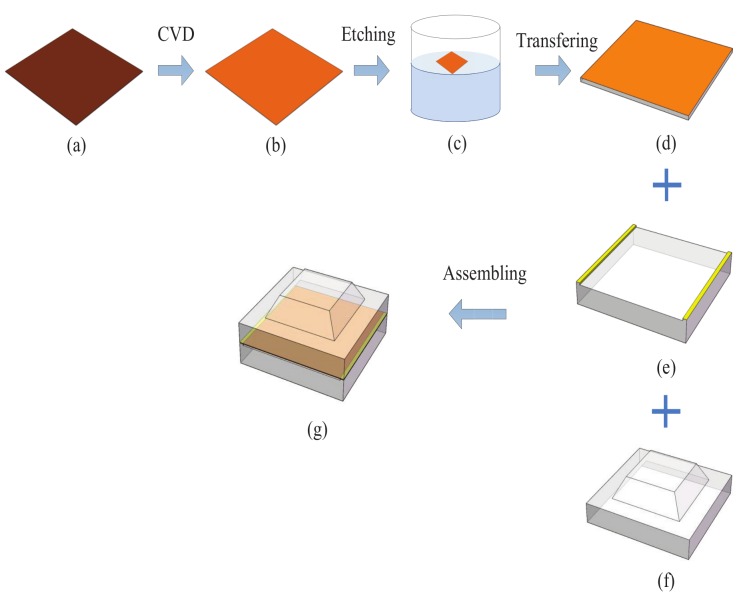
The assembling process of the sensor.

**Figure 5:**
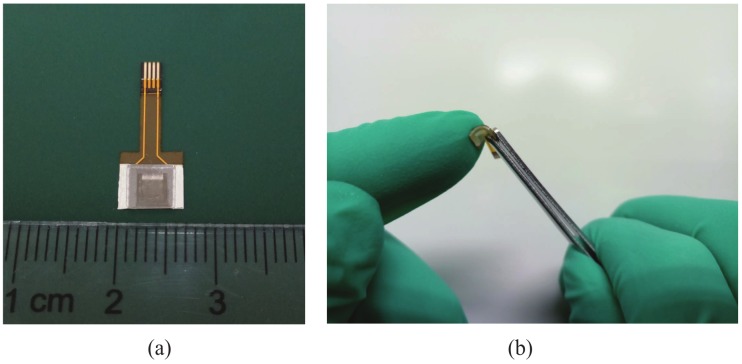
The photography of the flexible tactile sensor.

The graphene film was fabricated by Chemical vapor deposition (CVD) method and prepared by a wet transfer method. The specific fabrication processing of the flexible tactile sensor was shown as in Figure 4. Firstly, we fabricated a single-layer graphene film on a copper by CVD method (see Figure 4a). Secondly, we put the graphene-copper into a solution to etch off copper (see Figure 4c). Then we transferred the graphene on a PET film(see Figure 4d) and pasted them on the PI with the silver, which fabricated the electrodes on a PI (see Figure 4e).At last, we assembled the upper PDMS substrate with the Graphene/PET/FPCB film and the sensor was fabricated (see Figure 4g). The sample devices of the sensor was almost transparent and performed an excellent flexibility with a size of 5 mm × 7 mm × 1 mm (see Figure 5).

## 4. Experimental

**Figure 6:**
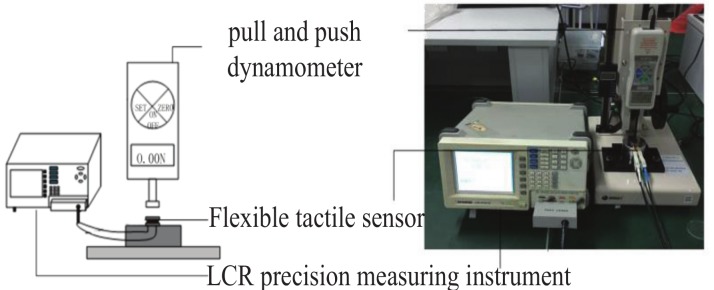
Experiment setup.

To test the performance of the sensor, we designed a micro-pressure testing platform (see Figure 6) which composed of a micro-force gauge (F1128 ZQ-20A-2, ZhiQu Co., Hangzhou, China) and a Lenz Capacitance and Resistance Digital Bridge Measuring Instrument (LCR-8101G, GWINSTEK, Taiwan). The micro-force gauge is able to applied a range of micro-force with a precision of 0.001N. The LCR Instrument is able to measure and record the output resistance of the sensor with a precision of 0.01 Ω. The sample devices of the sensor were placed on the testing bed of the micro-force gauge and connected with the LCR Instrument. When a micro-force was applied on the surface of the sensor by the micro-force gauge, the output resistance of the sensor was displayed and recorded by the LCR instrument.

## 5. Results and Discussion

### 5.1. Performance

**Figure 7:**
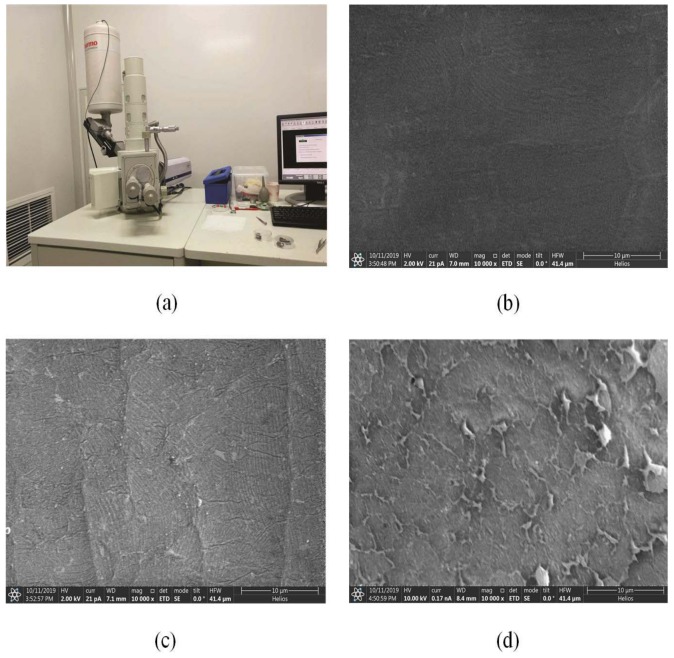
The sensor was scanned by the scanning electron microscope (**a**), and the micro-morphology of the graphene film of the sensor before pressed (**b**), after small pressed (**c**) and large pressure (**d**) were observed.

To validate the working principle of the sensor, we observed the micro-morphology of the sensor by the scanning electron microscope(SEM, JSM-6360LV, Co.JEOL, Tokyo, Japan), see Figure 7a. The micro-morphology of the graphene film of the sensor before pressed and after pressed are shown as in Figure 7b–d. Compared with these micro-morphology, we found that the number of crack in the graphene film grew with the increase of pressure applied on the sensor(see Figure 7c,d). The increasing crack will make the resistivity of the graphene film increased and in turns causing the output resistance of the sensor varied with the applied pressure.

**Figure 8:**
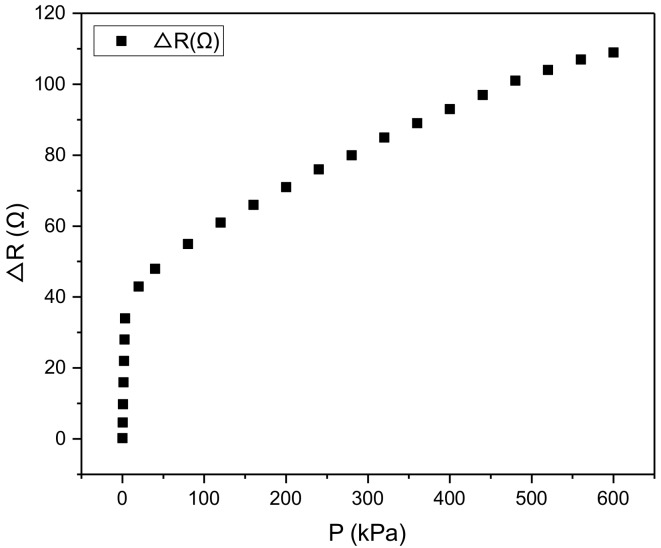
The sensor performed an ultra sensitivity at the range of 0–4 kPa and a large measurement range up to 600 kPa.

To test the performance of the sensor, we applied the pressure to the sensor and the result is shown as in Figure 8. From the figure we know that the relationship between the output resistance of the sensor and the applied pressure can be fitted by two straight line which are

(1) $$∆R\left(p\right)=\left\{\begin{array}{c} 10.56p+1.24,\;p\in [0,4\;kPa]\\ 0.11p+44.06,\;p\in [4\;kPa,600\;kPa] \end{array}\right.$$

From Equation (1), we know that the applied pressure can be measured according to the output resistance of the sensor. The sensor is able to measure a large range of 0–600 kPa pressure with a high sensitivity.

### 5.2. Sensitivity

Sensitivity is the ratio of the increment of the output to the increment of the input when the measurement system is performing a static measurement, which is

(2) $$S=\lim_{∆x\to 0}\left(\frac{∆y}{∆x}\right)=\frac{d_{y}}{d_{x}}$$

For a linear measurement system, the sensitivity is

(3) $$S=\frac{y}{x}=K=\frac{m_{y}}{m_{x}}\tan\theta$$

where $m_{x}$ and $m_{y}$ are the scale of the *x*-axis and *y*-axis respectively. θ is the angle between the tangent of the corresponding point and the *x*-axis. In other words, the sensitivity of the linear measurement system is a constant and can be obtained from the slope of the static characteristic curve.

In this work, we use the relative varies of the output resistance (∆R) as the *y*-axis and the applied pressure (P) as the *x*-axis of the static characteristic curve, i.e., ∆*R*-P curve. Therefore, the sensitivity of the sensor can be written by

(4) $$S=\frac{∆R}{P}$$

where ∆*R* is the output varies of the sensor (the unit is Ω). *P* is the applied pressure (the unit is kPa). Therefore, the unit of the sensitivity is Ω/kPa. From Equation (4), we know that the output resistance varies from 0 to 42 Ω at the range of 0–4 kPa, therefore, we obtain that

(5) $$S_{1}=\frac{41.8}{4-0.004}=10.56\;Ω/kPa$$

The sensitivity of the sensor at the range of 4–600 kPa is

(6) $$S_{2}=\frac{\left|109-41.8\right|}{600-4}=0.113\;Ω/kPa$$

Therefore, we concluded that the sensor have a high sensitivity of 10.56 Ω/kPa at the range of ∼4 kPa and a sensitivity of 0.113 Ω/kPa at the range of 4–6 kPa.

### 5.3. Linearity

Linearity is the degree of deviation between the actual input-output relationship and the fitting input-output relationship of a sensor. It is usually expressed by the maximum nonlinear error, which is

(7) $$\delta_{L}=\frac{{∆L}_{max}}{Y_{FS}}\times 100\%$$

where $\delta_{L}$ is the linearity; ${∆L}_{max}$ is the maximum deviation between the calibration line and the fitted line; $Y_{FS}$ is the full scale of the measurement range. In this work, we used the least squares method to fit the line. Therefore, the linearity refers to the least squares linearity.

**Figure 9:**
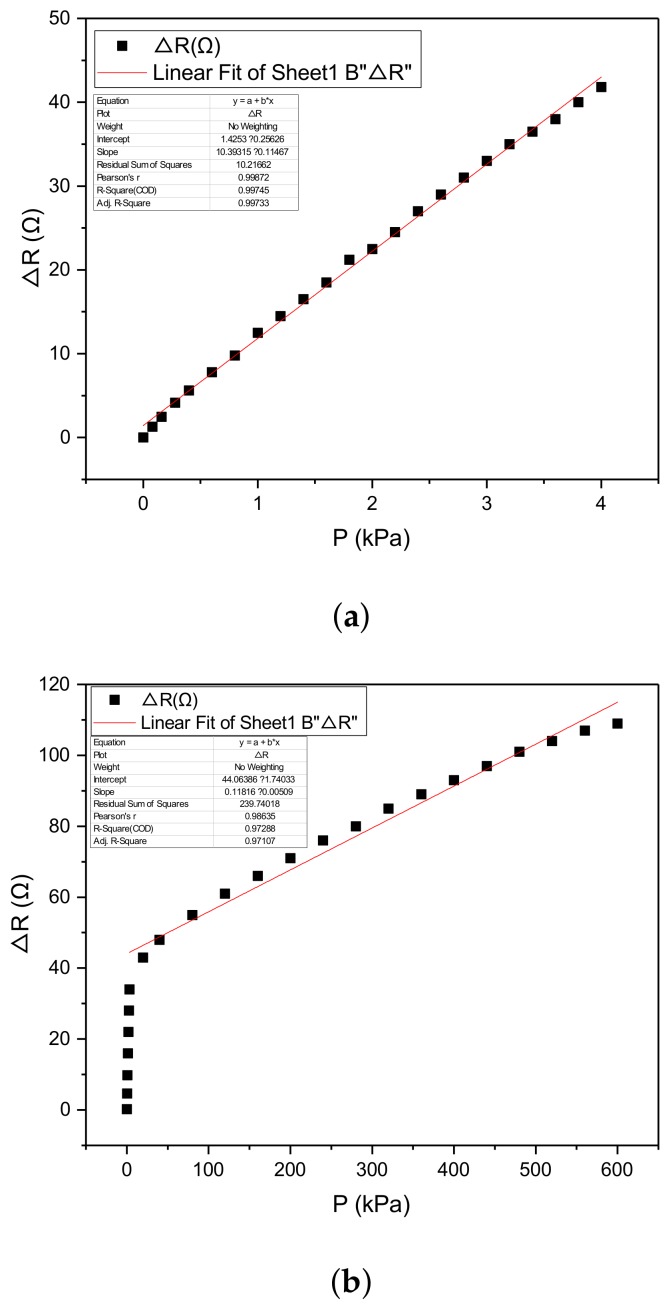
The linearity of the sensor is 3.28% at the range of 0–4 kPa (**a**) and 4.17% at the range of 4–600 kPa (**b**).

From Figure 9a we obtain that the linearity of the sensor presented in this work at the range of 0–4 kPa can be given by

(8) $$\delta_{L1}=\frac{\left|21.2-19.8\right|}{42.6}\times 100\%=3.28\%$$

The linearity of the sensor presented in this work at the range of 4–600 kPa can be given by

(9) $$\delta_{L2}=\frac{\left|72.5-67.7\right|}{115}\times 100\%=4.17\%$$

From Equations (8) and (9) we know that the sensor performed a better linearity at the low range of less than 4 kPa and a well linearity at the large range up to 600 kPa. These two linear input-output curve makes the sensor has an ultra-high sensitivity as well as has a large measurement range.

### 5.4. Hysteresis

Hysteresis refers to the phenomenon in which the input-output curve of the sensor is not coincident between the positive stroke and negitive stroke. It usually expressed by the maximum hysteresis error, which is

(10) $$\delta_{H}=\frac{|∆H_{max}|}{Y_{FS}}\times 100\%$$

where $\delta_{H}$ is the maximum hysteresis error; ${∆H}_{max}$ is the maximum deviation between the positive and the negative stroke; $Y_{FS}$ is the full scale of the measurement range.

**Figure 10:**
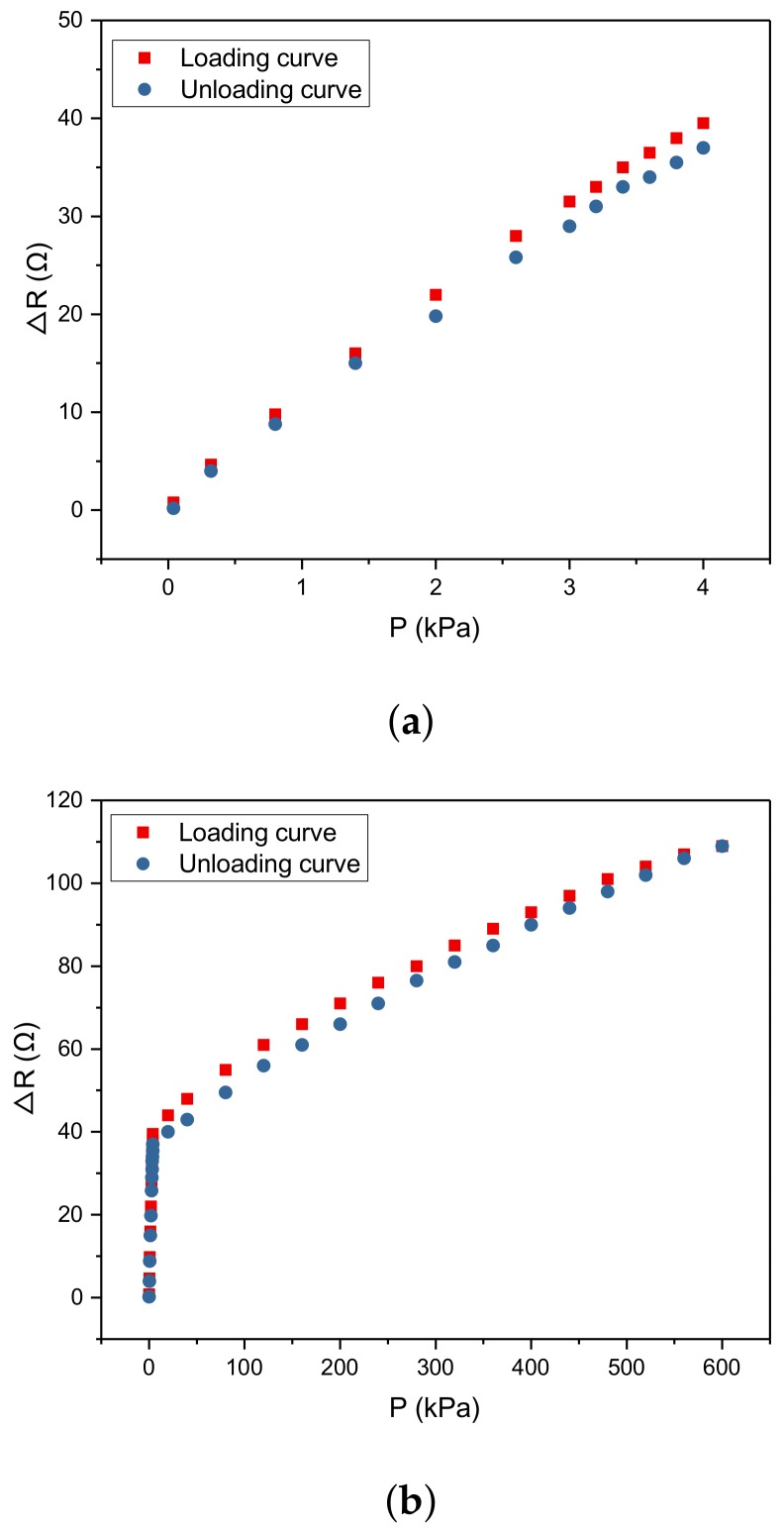
The hysteresis of the sensor is 5.87% at the range of 0–4 kPa (**a**) and 5.52% at the range of 4–600 kPa (**b**).

In this work, the hysteresis curve of the sensor is shown as in Figure 10a. According to Figure 10a, we obtain that the hysteresis error of the sensor at the range of 0–4 kPa can be given by

(11) $$\delta_{H}=\frac{\left|39.5-37\right|}{42.6}\times 100\%=5.87\%$$

The hysteresis error of the sensor at the range of 4–600 kPa can be given by

(12) $$\delta_{H}=\frac{\left|44-40\right|}{115-42.6}\times 100\%=5.52\%$$

### 5.5. Repeatability

Repeatability is the inconsistency of the characteristic curves when the same sensor is tested under the same working conditions for multiple executions of the experiment. It is expressed by repeatability error which is given by

(13) $$\delta_{R}=\frac{|Z\delta_{max}|}{Y_{FS}}\times 100\%$$

where $\delta_{R}$ is the repeatability error. *Z* is the confidence coefficient. For the normal distribution, the confidence probability is 99.73% when *Z* equals 3. $\delta_{max}$ is the maximum value of the standard deviation of each measurement point. $Y_{FS}$ is the full-scale of the sensor.

The standard deviation $\delta_{max}$ can be calculated by the Bessel Formula, which is

(14) $$\delta_{max}=max\left(\delta \right)=\sqrt{\frac{1}{N-1}\sum_{i=1}^{N}{\left(R_{i}-\bar{R}\right)}^{2}}$$

where *i* is the index of the measurement point; $R_{i}$ is the corresponding measurement and $\bar{R}$ is the average value of the measurement points.

**Figure 11:**
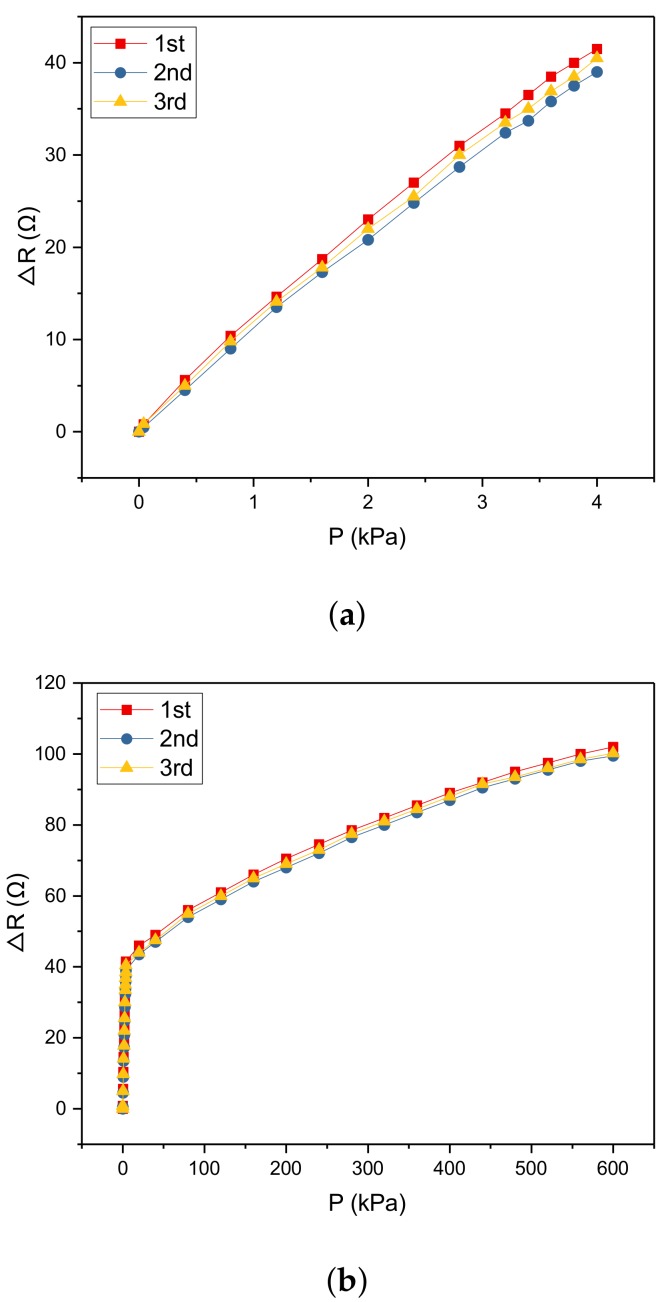
The repeatability of the sensor is 5.87% at the range of 0–4 kPa (**a**) and 5.52% at the range of 4–600 kPa (**b**).

In this work, the hysteresis curve of the sensor is shown as in Figure 11. According to the figure, we obtain that the hysteresis of the sensor at the range of 0–4 kPa can be given by

(15) $$\delta_{H_{1}}=\frac{{∆H}_{max}}{Y_{FS}}\times 100\%=\frac{\left|39.5-37\right|}{42.6}\times 100\%=5.87\%$$

The hysteresis of the sensor at the range of 4–600 kPa can be given by

(16) $$\delta_{H_{2}}=\frac{{∆H}_{max}}{Y_{FS}}\times 100\%=\frac{\left|44-40\right|}{115-42.6}\times 100\%=5.52\%$$

**Figure 12:**
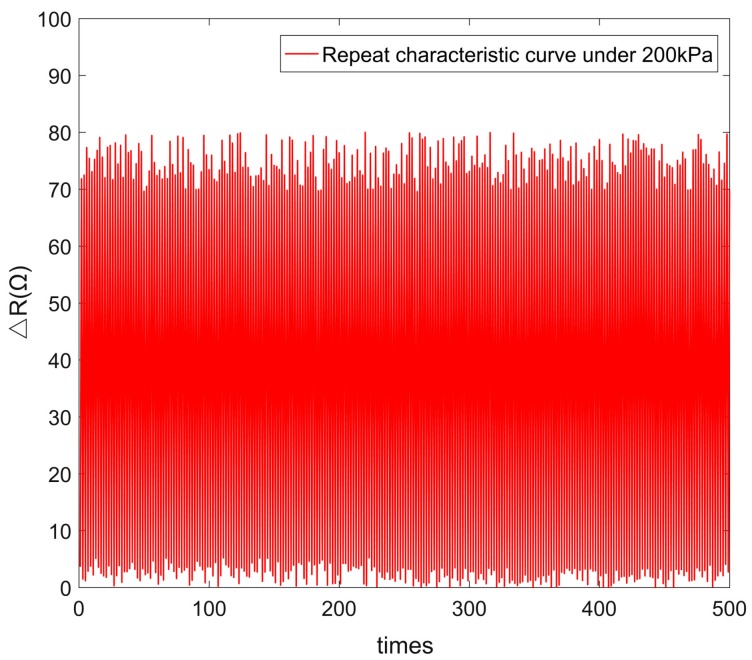
The cycling test of the tactile sensor applied with a force of 200 kPa.

The cycling test of the fabricated tactile sensor is carried out by loading a force of 200 kPa. Results showed that the sensor has good repeatability during 500 cycles, as shown in Figure 12.

### 5.6. Dynamic Performance

The dynamic characteristic refers to the response time after the sensor’s input changes. The response time is the delay time between the output and corresponding input. For a tactile sensor, the dynamic performance refers to the load and unload times of the sensor.

**Figure 13:**
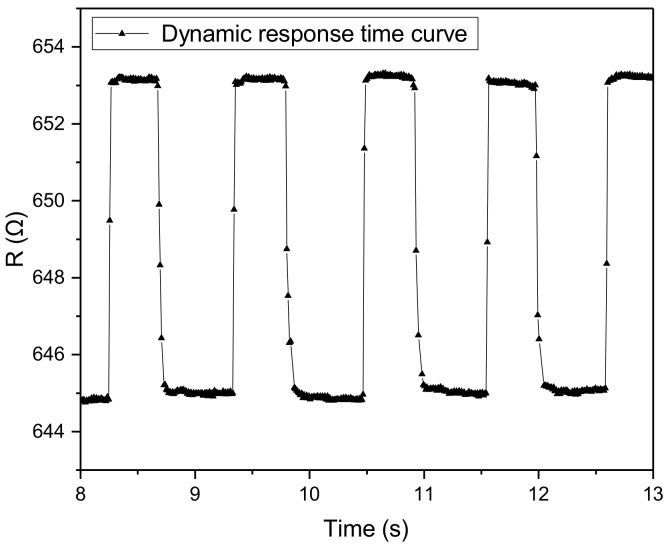
The sensor is based on a PI substrate and four Ni thermal element.

In this work, we load a force of 0.02 N to the sensor repeatability and record the output capacitance of the sensor by a source meter (Keithley 2450, Tektronix, Beaverton, OR, USA) with a sampling period of 10 ms. The dynamic characteristic of the sensor is shown as in Figure 13. From the figure, we obtain that the loading time and the unloading time of the sensor is 10 ms and 30 ms, respectively.

### 5.7. Flexibility

**Figure 14:**
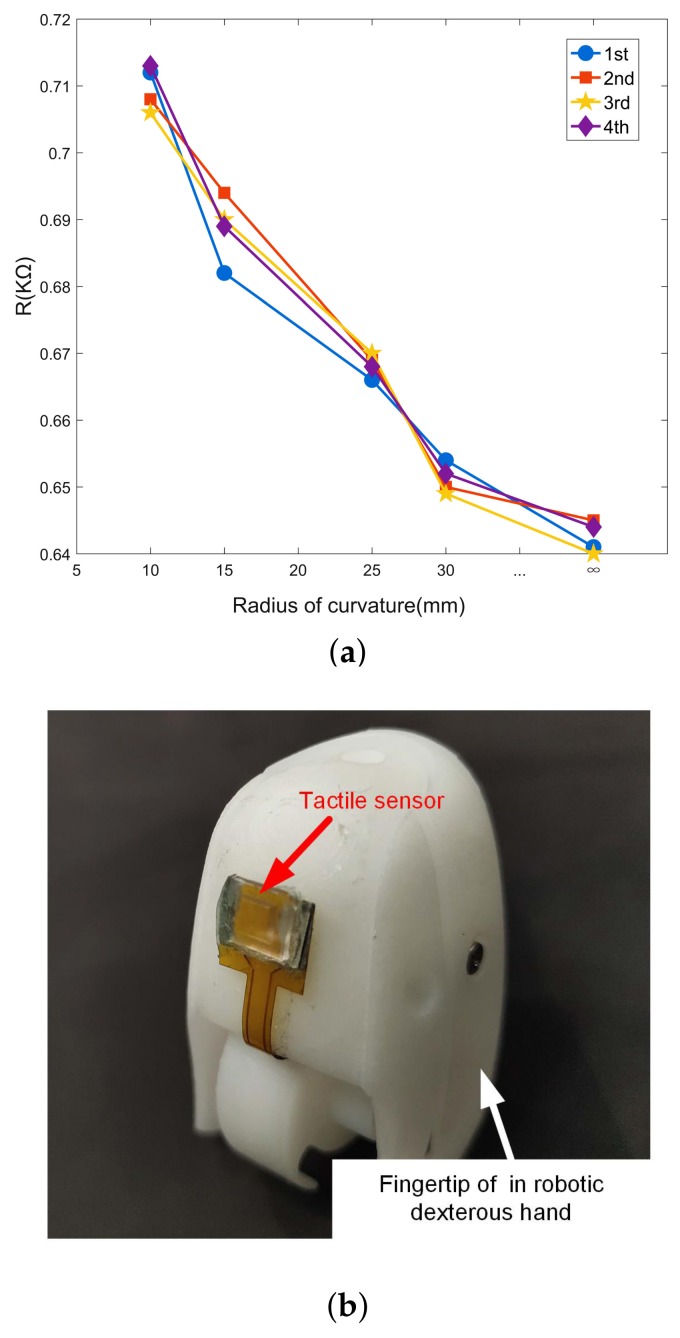
The flexility of the sensor: (**a**) cycling test under force of 200 kPa and (**b**) mounted on the fingertip of robotic dexterous hand.

The flexible property of fabricated tactile sensor are tested on the curved surface with radius of curvature from 10 mm to 30 mm. Results showed that the tactile sensor features good flexibility and repeatability with various radius of curvature. As shown in Figure 14a, with the decrease of the radius of curvature of the curved surface, the initial resistance of the sensor mounted reduces slowly. Therefore, the proposed flexible sensor can be utilized for fingertip of robotic dexterous hand to provide haptic perception, as shown in Figure 14b.

### 5.8. Sensors Comparisone

To compare our work with the state of the art, we given a table to list the material, sensitivity, precision, measurement range, span scale, response time (RT) and the size of the references sensors (see Table 1). The span scale is the ratio between the minimum resolution and maximum measurement range of the sensor. For a tactile sensor, a better span scale means the sensor has a good minimum resolution as well as has a large measurement range. In this work, the span scale of our presented sensor equals 4, which means there are 4 orders magnitude between the minimum resolution and maximum measurement range. This result increased by 1–2 order of magnitude compared with other refereces (2–3 orders of magnitudes). From the table, we also know that the sensor of our work have excellent performance in precision(0.004 kPa), large measurement range(600 kPa), high response time(10 ms) and small size(5 mm × 7 mm).

## 6. Conclusions

In this work, we presented a ultra-sensitivity flexible tactile sensor based on graphene film. The sensor is based on piezoresistive effect of graphene film and is composed of upper substrate (PDMS with a thickness of 5 μ m and a bump thickness of 1 mm), medial graphene film (graphene film with a size of 5 mm × 7 mm) and lower substrate (PET with a hardness of 2H). We fabricated sample sensor devices and carried out experiment to test the performance. According to the result, we concluded that the sensor is able to measure the pressure at the range of 0–600 kPa. The sensor has an ultra-sensitivity of 10.56 Ω/kPa at the range of 0–4 kPa with a linearity error, hysteresis error and repeatability of 3.28%, 5.87% and 4.92% respectively. The sensor has a sensitivity of 0.113 Ω/kPa at the range of 4–600 kPa with a linearity error, hysteresis error and repeatability of 4.17%, 5.52% and 4.79% respectively. The sensor performs an excellent dynamic characteristic of loading time of 10 ms and unloading time of 30 ms. The sensor also has 4 orders of magnitude between minimum resolution and maximum measurement range which have great advantage compared with state of the art. The sensor is expected have great application prospect in robot and biomedical.

## Figures and Tables

**Table 1:** Sensors comparison.

Ref.	Material	Sensitivity (kPa${}^{-1}$)	Precision (kPa)	Measuring Range (kPa)	Span Scale	RT (ms)	Size (mm)
[12]	AgNWs/rGO	5.8	0.000125	0–0.1	2	29.5	20×1×1
[16]	CNT/PDMS	0.0173	0.128	0.128–44	2	-	15×15×5
[17]	ITO/SSNPs-PU/ITO	2.46	0.3	0.3–24.5	1	30	-
[18]	rGO	−5.53	0.0015	0-1	2	0.2	>100×100
[19]	CNTs/CB/SR	> 0.04	0.1N	0–1562.5 (0–100 N)	3	-	R = 4
Our work	GR	0.04	0.004	0–600	4	10	5×5×5
